# The use of protein phosphatase 2A activators in combination therapies for pancreas cancer

**DOI:** 10.18632/oncotarget.26772

**Published:** 2019-03-12

**Authors:** Brittany L. Allen-Petersen, Rosalie C. Sears

**Affiliations:** Rosalie C. Sears: Department of Molecular and Medical Genetics, Oregon Health and Science University, Portland, OR, USA

**Keywords:** PP2A, MYC, pancreatic cancer

Pancreatic adenocarcinoma (PDA) is extremely aggressive, often discovered late in disease progression, and associated with extremely high mortality rates. While targeted therapies have shown great promise in cancers such as breast and chronic myeloid leukemia, these agents have shown little efficacy in PDA patients. The underlying reasons for this therapeutic resistance is highly debated in the field. From a therapeutic standpoint, the primary target for PDA tumors is oncogenic KRAS, which is almost universally mutated in PDA patients, but considered to be “undruggable.” Additionally, several reports indicate that mutant KRAS makes PDA cells excellent metabolic scavengers, suppresses immune surveillance, and results in a high degree of signaling plasticity that can circumvent single agent treatment [1-3]. Many researchers are now advocating for combination strategies as a means to prevent signaling adaptation and increase therapeutic efficacy in PDA patients. Several therapeutic agents show synergistic activity in combination either with other targeted agents, checkpoint inhibitors, or chemotherapies, highlighting the potential of these strategies for the treatment of this therapeutically refractory disease [4].

Protein phosphatase 2A (PP2A), a major serine/threonine phosphatase, is a critical regulator of transformation, decreasing the activation of proteins downstream of oncogenic KRAS, including ERK and mTOR. PP2A is aberrantly deactivated in cancer cells, indicating that PP2A suppression may be a common event in cancer progression. Recent work from Kauko et. al. suggests that PP2A regulates an extensive number of cancer related pathways and that the loss of PP2A results in increased therapeutic resistance to over 200 compounds [5]. Conversely, the inhibition of SET, an endogenous PP2A inhibitor, increased the overall therapeutic sensitivity of cells to targeted agents, placing PP2A as a central mediator of therapeutic response. Consistent with the role of PP2A suppression in cancer, therapeutic agents that increase the activity of PP2A have shown excellent anti-tumor responses in pancreas, breast, ovarian, prostate, and lung cancer. Importantly, these agents induce death specifically in cancer cells, with little to no *in vivo* toxicity, uncovering a cancer cell signaling dependency on PP2A inhibition [6].

Recently, we reported that PP2A activating agents could function synergistically with kinase inhibitors to induce cell death in PDA cells [7]. Similar to SET knockdown, the activation of PP2A reduced the IC50 of multiple therapeutic agents within a kinase inhibitor screen, with inhibitors to EGFR/HER2, Aurora kinases, Src family kinases, and PI3K/mTOR displaying the strongest synergy. Direct PP2A activation, through the use of a Small Molecule Activator of PP2A (SMAP), combined with the mTOR inhibitor, INK128, resulted in a synergistic reduction in tumorigenic phenotypes both *in vitro* and *in vivo*. In addition to the loss of the AKT/mTOR pathways, this combination also significantly reduced the PP2A target, c-MYC (MYC). MYC is a potent transcription factor that regulates a wide range of cellular functions and significantly contributes to tumor aggressiveness and therapeutic resistance, making therapeutics that target MYC highly desirable [8, 9]. PP2A has been shown to dephosphorylate MYC, decreasing its activity and stability, suggesting that altered MYC signaling may significantly contribute to the efficacy of our combination strategy [10]. Consistent with this hypothesis, we demonstrated that aberrant expression of MYC increased resistance to mTOR inhibition. These results indicate that loss of AKT/mTOR signaling alone may not be sufficient to drive significant cell death, and highlights the importance of incorporating compounds that decrease MYC activity into combination strategies.

While these studies support the use of SMAPs to reduce MYC activity and suppress signaling plasticity to enhance the efficacy of targeted therapeutics, several questions remain. PP2A activity is modulated through multiple mechanisms, including association with inhibitory partners, such as SET and CIP2A, and posttranslational modifications to PP2A subunits. What is the biologic consequence and therapeutic implications of these different mechanisms of PP2A regulation on cancer phenotypes? Further, are there specific modes of PP2A inhibition that are more strongly associated with the ability of cancer cells to maintain MYC levels? Finally, would indirect or direct PP2A activators show preferential activity in patients and could the combination of both methods lead to a more complete loss of oncogenic signaling?

We also found that in certain PDA cell lines neither mTOR inhibition nor SMAP-mediated PP2A activation was able to decrease the MYC phosphorylation levels. Interestingly, these cell lines are of the quasi-mesenchymal (QM) PDA subtype, which is characterized by the enrichment of MYC driven pathways. Combined, these data indicate that there may be specific PDA subtypes that are susceptible to the combination of mTOR inhibition and PP2A activation. It is possible that QM cell lines acquire unique or redundant mechanisms to maintain aberrant MYC signaling, allowing these cells to become less responsive to mTOR inhibitors. A mechanistic understanding of the relationship between PP2A and MYC will help identify patients that may benefit from the therapeutic activation of PP2A and may lead to new therapeutic combinations.

Given the critical role that phosphatases play in maintaining the balance between active and inactive signaling states, the therapeutic activation of PP2A has broad implications for many tumor types. Ultimately, understanding the mechanisms of cancer-associated PP2A inhibition and the functional consequence of this inhibition is of the utmost importance for identifying therapeutic resistance mechanisms and the clinical utility of PP2A activators.

## Figures and Tables

**Figure 1:**
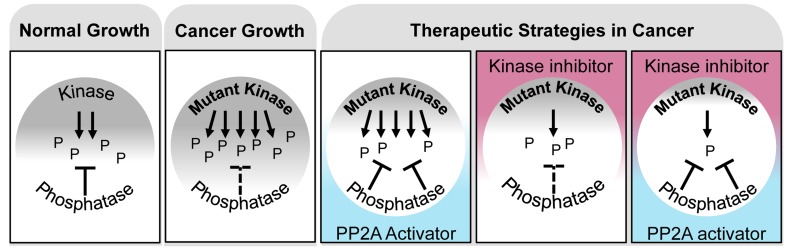
PP2A activation and kinase inhibition as a novel therapeutic combination strategy in cancer During normal cell growth there exists a critical balance between kinases and phosphatases in order to maintain signaling homeostasis (**Normal Growth**). In cancer, genetic mutations disrupt this balance, resulting in aberrant phosphorylation through the activation of kinase cascades and the suppression of phosphatases (**Cancer Growth**). Therapeutic inhibition of kinases or activation of the phosphatase PP2A attenuates oncogenic cascades; however, the combination of these two strategies results in a synergistic loss of cancer cell viability (**Therapeutic Strategies in Cancer**). P = phosphorylation
